# Insight into the Inclusion Complexation of Fluconazole with Sulfonatocalix[4]naphthalene in Aqueous Solution, Solid-State, and Its Antimycotic Activity

**DOI:** 10.3390/molecules27144425

**Published:** 2022-07-11

**Authors:** Tayel A. Al Hujran, Mousa K. Magharbeh, Almeqdad Y. Habashneh, Rasha S. Al-Dmour, Ashraf Aboelela, Hesham M. Tawfeek

**Affiliations:** 1 The Department of Pharmaceutical Chemistry, Faculty of Pharmacy, Mutah University, Al-Karak 61710, Jordan; magharbeh@mutah.edu.jo (M.K.M.); rasha.aldmour@mutah.edu.jo (R.S.A.-D.); 2 The Department of Chemistry, School of Science, The University of Jordan, Amman 11942, Jordan; a.habashneh@ju.edu.jo; 3 The Department of Pharmaceutical Chemistry, Faculty of Pharmacy, Sphinx University, Assiut 71515, Egypt; ashrafam@sphinx.edu.eg; 4 Industrial Pharmacy Department, Faculty of Pharmacy, Assiut University, Assiut 71526, Egypt; heshamtawfeek@aun.edu.eg

**Keywords:** antimycotic activity, dissolution rate, fluconazole, inclusion complexation, phase solubility, sulfonatocalix[4]naphthalene

## Abstract

The study aims to assess the interaction between fluconazole and sulfonatocalix[4]naphthalene towards enhancing its dissolution performance and antimycotic activity. A solubility study was carried out at different pH conditions, and the results revealed the formation of a 1:1 molar ratio fluconazole-sulfonatocalix[4]naphthalene inclusion complex with an A_L_ type phase solubility diagrams. The solid powder systems of fluconazole-sulfonatocalix[4]naphthalene were prepared using kneaded and co-evaporation techniques and physical mixtures. DCS, PXRD, TGA-DTG, FT-IR, and in vitro dissolution performance characterize the prepared systems. According to physicochemical characterization, the co-evaporation approach produces an amorphous inclusion complex of the drug inside the cavity of sulfonatocalix[4]naphthalene. The co-evaporate product significantly increased the drug dissolution rate up to 93 ± 1.77% within 10 min, unlike other prepared solid powders. The antimycotic activity showed an increase substantially (*p* ≤ 0.05, *t*-test) antimycotic activity of fluconazole co-evaporate mixture with sulfonatocalix[4]naphthalene compared with fluconazole alone against clinical strains of *Candida albicans* and *Candida glabrata*. In conclusion, sulfonatocalix[4]naphthalene could be considered an efficient complexing agent for fluconazole to enhance its aqueous solubility, dissolution performance, and antimycotic activity.

## 1. Introduction

Inclusion complexation between the host (macrocyclic molecules) and guest (drug) has attracted tremendous attention to improve the aqueous bioavailability and solubility of poor water-soluble drugs [1,2,3]. The cyclic oligosaccharide cyclodextrins (CDs) and their semisynthetic derivatives such as hydroxypropyl-*β*-CD and methyl-*β*-CD are the most valuable carriers in drug delivery as host receptors [1,2,4,5,6,7,8]. However, *β*-CD showed limited water solubility since, in a crystal state, they possess strong intermolecular hydrogen bonding, which prevents them from associating with the surrounding water molecules [9]. Hydroxypropyl-*β*-CD showed some hematological disturbance and GIT problems such as diarrhea upon dosing for up to three months [10]. Moreover, the modified derivatives showed a higher water solubility, as with *γ*-CD and sulfobutylether *β*-CD. Still, they are so expensive and need a lot of steps of functionalization and purification for their industrial scalability. This highlights the significance of other complexing agents in safe and economical drug delivery applications. Calixarenes, a group of water-soluble anionic macrocyclic compounds, have been developed and investigated [11]. However, the number of reported complexation studies of the calixarene with different drugs is still few compared with CDs.

**Figure 1:**
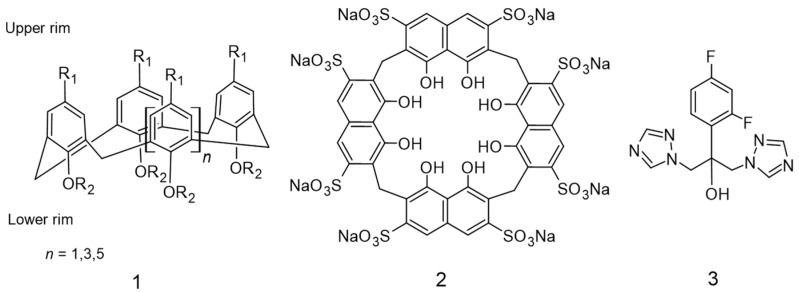
The structures of the calix[*n*]arene (1), sulfonatocalix[4]naphthalene (2), and fluconazole (3).

The first synthesized calixarenes have limited application due to their high hydrophobicity and poor aqueous solubility [12]. The hydrophobicity problem was solved by modifying the lower and upper rims of calixarenes macrocyclic via the introduction of some water-soluble ionizable groups, e.g., sulfonates [13], ammonium [12], phosphates [14], and carboxylates (Figure 1, compound **1**) [7,8,9,10,11]. This modification enhanced the hydrophilicity and could improve their complexation ability with different host drug compounds in the aqueous solutions [15,16,17]. Furthermore, calix[*n*]arenes exhibited better compatibility and decreased toxicity than naturally occurring macrocyclic CD’s, and their fast and easy synthesis method could be adapted for large-scale production [18,19]. For these advantages, the potential application of calix[*n*]arenes is reported to enhance the bioavailability and water solubility of different drugs such as oxaliplatin [20], doxorubicin [21], isoniazid ciprofloxacin [22], tenofovir disoproxil fumarate [23], carbamazepine [14], nifedipine [14,24], and niclosamide [14,24].

The sulfonatocalix[4]naphthalene macrocycle has unique properties, such as higher water solubility and large cavity size compared with CD’s and *p*-sulfonatocalix[*n*]arene macrocycles (Figure 1, compound **2**) [25,26,27,28]. We have earlier reported the complexation study of sulfonatocalix[4]naphthalene with meloxicam, which showed an improvement in water solubility and enhancement of the dissolution performance of the drug [29].

Fluconazole (FZ) is a synthetic bistriazole antifungal agent (Figure 1, compound **3**) [30,31]. FZ has a lipophilic moiety 2,4-difluorophenyl at position 2, and two triazole rings at positions 1 and 3 connected to the *tert*-alcohol (2-propanol), hydrophilic moiety [32]. It is considered the first line to treat a wide range of superficial and systematic fungal infections with *Candida* species [33] due to the lower side effects [34] and the reported low toxicity [30]. It is also prescribed for patients suffering from acquired immunodeficiency syndrome (AIDS) [35] and patients subjected to immunosuppressant therapies such as cancer and transplant patients acute leukemia [36], and bone marrow transplantation patients [37] to prevent cryptococcal meningitis, the non-*albicans candidiasis*, and yeast infections, respectively [34,38,39,40,41]. FZ’s mechanism of action inhibits producing the major fungal cell membrane lipid ergosterol [34,42]. According to the biopharmaceutical classification system (BCS), FZ belongs to classes I and III [43]. The poorly soluble drugs suffer from lower bioavailability, consequently, lower absorbance by the gastrointestinal tract (GIT). This limitation is overcome by developing various techniques to improve the physicochemical properties of those types of drugs such as solid dispersions [44], incorporation into lipid vesicles [45,46], microemulsions [47,48], micellar solubilization [49], particle size reduction [50], cocrystals [51], and complexation [41,52,53].

Lower water solubility is among the significant pharmaceutical problems related to FZ, estimated at room temperature to be around 1.0 mg/L [42,54]. Moreover, the low aqueous solubility of the drug could affect its bioavailability and therapeutic performance [34]. Different research groups reported many complexation studies of the FZ with varying types of naturally occurring and semisynthetic macrocyclic cyclodextrins [38,48,55,56,57,58]. Abranches et al. [34] reported a complexation study of FZ with sodium *p*-sulfonatocalix[*n*]arenes (*n* = 4 and 6). They found the inclusion complexation affected by the cavity size of the host [34]. Therefore, the present study assesses FZ’s possible interaction and complexation with highly water-soluble and deep-large cavity size calix[*n*]arene derivative.

The complexation ability of FZ with sulfonatocalix[4]naphthalene at both aqueous solutions and in the solid-state was investigated and studied through the construction of phase solubility diagrams in distilled water and different pH conditions. Furthermore, the prepared solid powders via kneading and solvent evaporation were characterized via DSC, TGA, DTG, PXRD, and FT-IR to evaluate the interaction between FZ and the host molecule. In vitro dissolution performances were also studied and analyzed for relative dissolution rate and efficiency. Finally, two different pathogenic strains, *Candida glabrata,* and *Candida albicans* were used to perform the antimycotic activity.

## 2. Results and Discussion

### 2.1. Phase Solubility Test

**Figure 2:**
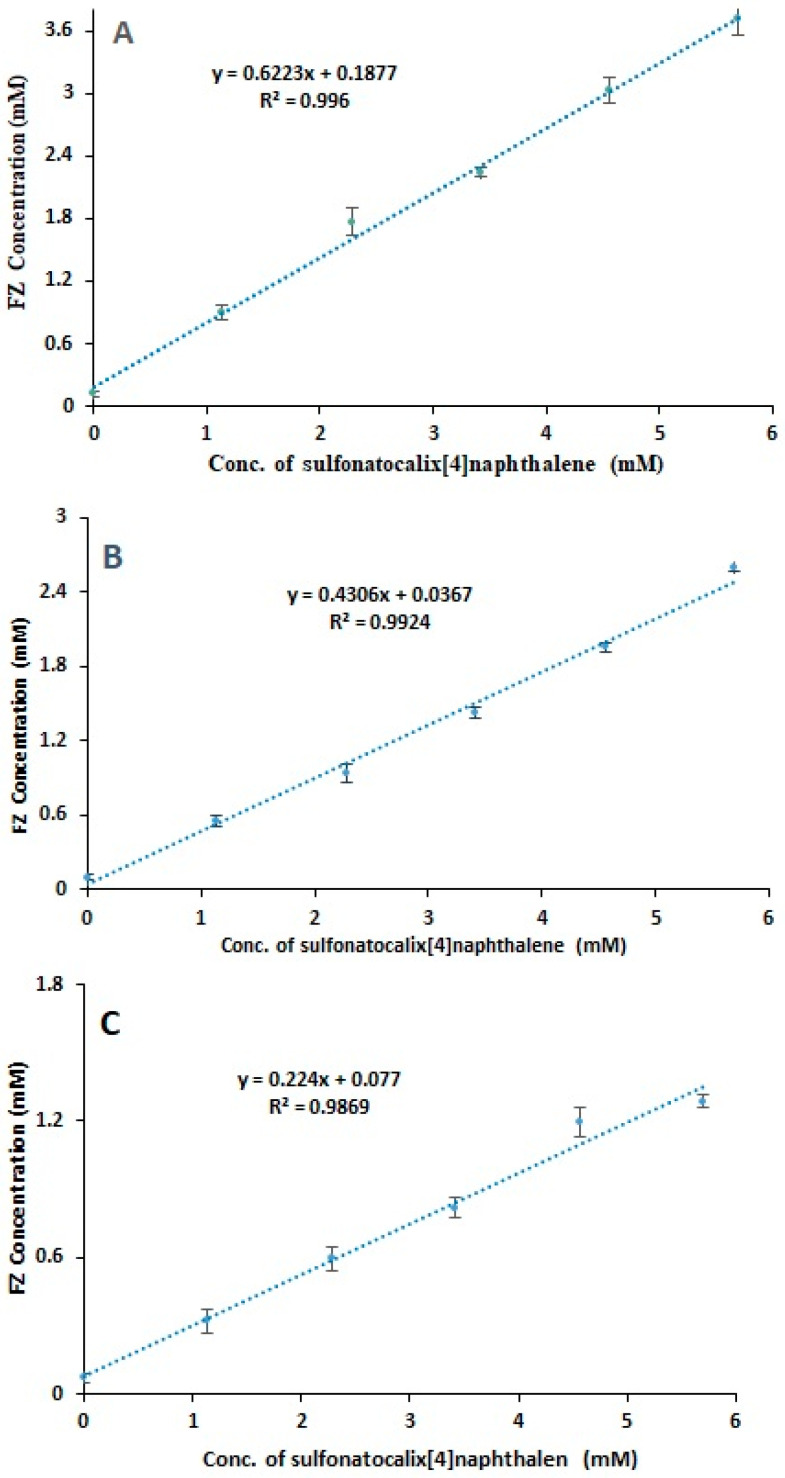
Phase solubility diagrams of FZ in the presence of different concentrations of sulfonatecalix[4]naphthalene at temp. 25.5 ± 0.5 °C. (**A**) pH 1.2, (**B**) phosphate buffer pH 7.4, (**C**) distilled water. (*n* = 3, ±S.D.).

FZ’s oral bioavailability is exceedingly low due to its poor solubility in an aqueous solution, making solubility and dissolution rate-determining processes in drug absorption [59]. The method described by Higuchi and Connors [57] was used to conduct the phase solubility study of FZ. It was revealed that the FZ’s solubility was markedly increased when sulfonatocalix[4]naphthalene was present. The phase solubility was presented by plotting equilibrium concentrations of the FZ against different concentrations of sulfonatocalix[4]naphthalene (Figure 2). It was found that there was a linear increase in FZ’s solubility with increasing concentration of sulfonatocalix[4]naphthalene at the investigated different pH media, compared with FZ alone. The obtained phase solubility diagrams had a slope value of less than one, delineating an A_L_ type complex. The enhancement of FZ solubility might be attributable to water-soluble FZ-sulfonatocalix[4]naphthalene inclusion complex formation by hosting the FZ in a 1:1 molar ratio. The weak intermolecular interaction forces including H**^…^**F hydrogen bonding, *π-π* interactions, dipole-dipole interaction, or electrostatic interaction between hydroxyl groups and hydrophobic cavity of sulfonatecalix[4]naphthalene and drug aromatic ring, or other functional groups of the drug molecule are strongly recommended the complex formation in the aqueous state [34,60,61].

The linear regression analysis of the obtained phase solubility diagrams estimated stability constants (Ks) of the FZ-sulfonatocalix[4]naphthalene complex (1:1 molar ratio) at 25 ± 0.5 °C, which were in the rank order of 2861.112 ± 0.095, 1881.830 ± 0.012, 501.266 ± 0.011 M^−1^ for pH 1.2, distilled water, and phosphate buffer of pH 7.4; respectively (Table 1). Also, FZ solubility increased 31-folds at pH 1.2 and 25-folds at pH 7.4, attributing to the weak acid-base nature of the FZ, where the first protonation triazole rings occur around pKa = 2.56 ± 0.12, and the second at pKa = 2.94 ± 0.1 and deprotonation the hydroxyl group above pKa = 11.01 ± 0.29 [30]. FZ has a greater solubility in both acidic and basic environments. This result is also consistent with the reported findings of Auti et al. [62]. However, creating the FZ-sulfonatocalix[4]naphthalene complex in an aqueous medium increased its water solubility in acidic and basic medium, which would eventually increase its dissolution and bioavailability, resulting in enhanced antifungal activity.

### 2.2. Physicochemical Characterization for the Conformation of Inclusion Complex

#### 2.2.1. Differential Scanning Calorimetry (DSC)

**Figure 3:**
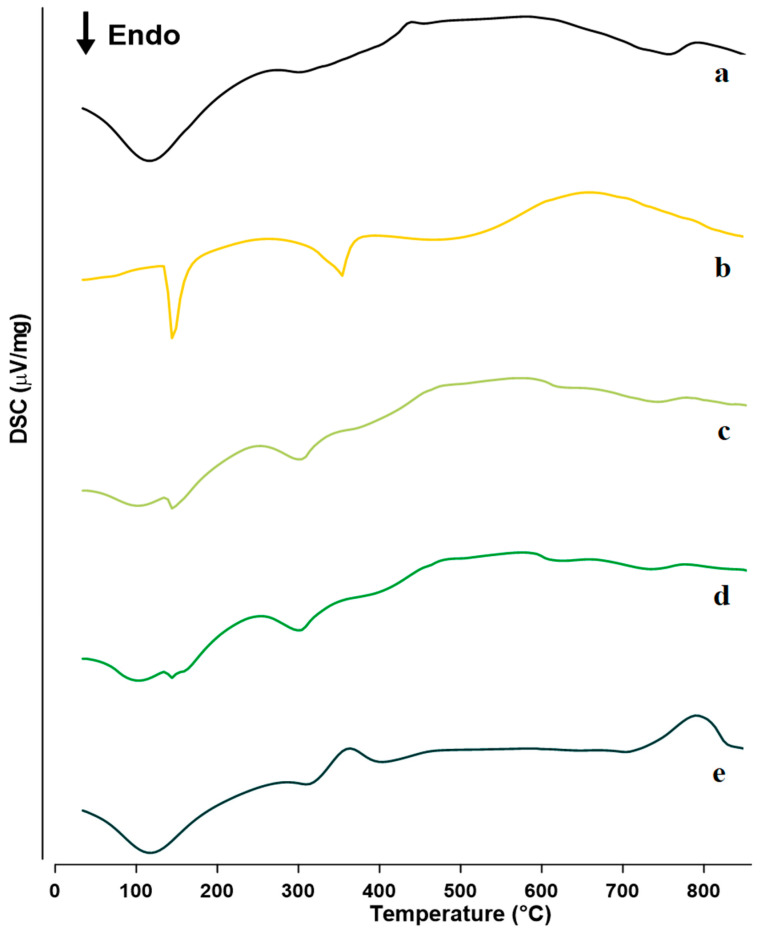
DSC thermograms for: (**a**) Sulfonatocalix[4]naphthalene, (**b**) fluconazole (FZ) (**c**) Physical mixture (PM), (**d**) kneaded (KN), and (**e**) co-evaporate powder (CO).

Thermal analysis is a reliable approach for identifying and characterizing the development of inclusion complexes between medicines and their carriers and assessing their thermal stability [42,63]. Furthermore, DSC is the most extensively used method for recognizing amorphous phases in multi-component systems. Moreover, it is a straightforward and quick way for determining the compatibility of various medications with excipients [55,64]. The thermal behavior of FZ, sulfonatocalix[4]naphthalene, solid complexes, and physical mixtures were investigated for the possibility of complexation and contact. The DSC thermograms of sulfonatocalix[4]naphthalene, untreated FZ, and produced solid systems are shown in Figure 3. The DSC thermal analysis curves for the sulfonatocalix[4]naphthalene, physical, kneaded, and co-evaporate solid powders showed an endothermic dehydration peak around 108 °C. The pure FZ thermogram (Figure 3b) revealed a single strong endothermic melting peak at roughly 140.0 °C, which correlates to its claimed melting temperature [42,55]. 

The DSC thermogram of sulfonatocalix[4]naphthalene showed two major endothermic events at 108.0 °C and 750.0 °C, attributed to its dehydration and fusion or decomposition [29]; respectively. The results showing the decreased intensity of the endothermic melting point peak of FZ and shifted to the lower temperature around 137.9 °C and 137.0 °C, respectively, were revealed through thermograms of the physical and kneaded powder products of the drug host. This result could be due to the lowering of FZ crystallinity due to mixing with amorphous sulfonatocalix[4]naphthalene, and the formation of a partial inclusion complex or the presence of FZ is an ultrafine crystallite [29,42].

While in the case of a co-evaporate mixture, no endothermic peak was observed. This result suggested the formation of an amorphous product and complete inclusion complexes between FZ drug and sulfonatocalix[4]naphthalene. A similar finding was previously reported [36,47], which confirmed the formation of an inclusion complex. Much more information about the possibility of the carrier converting the FZ physical state from crystalline to partially or completely amorphization will be investigated using TGA, PXRD, and FT-IR analytical tools.

#### 2.2.2. Thermogravimetric Analysis (TGA)

TGA is an important and widely used method for distinguishing various amorphous materials, including polymer materials and drug inclusion complexes [63,65]. TGA was used to study the thermal stability of physical, kneaded, and co-evaporate solid systems of FZ and sulfonatocalix[4]naphthalene. According to the TGA-DTG thermogram study, FZ begins to degrade at 350.0 °C with a mass loss of 99.34% in a single exothermic event. The sulfonatocalix[4]naphthalene TGA-DTG thermogram showed several thermal events. The first one is the dehydration endothermic event that occurred at 112.0 °C with a 15.2% loss of water between 65.0 °C and 165.0 °C, and the other two exothermic degradation events were observed at the higher temperature of 425.0 °C and 729.0 °C with 23.99% and 17.02% loss of mass; respectively.

**Figure 4:**
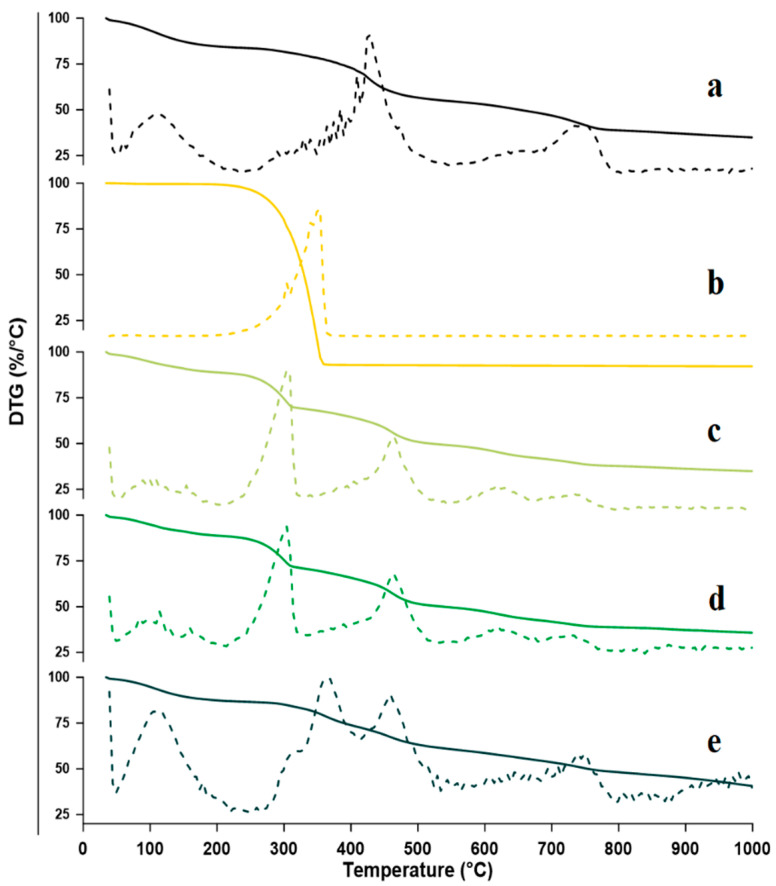
TGA/DTG curves for: (**a**) Sulfonatocalix[4]naphthalene, (**b**) fluconazole (FZ) (**c**) Physical mixture (PM) (**d**) Kneaded (KN) and (**e**) co-evaporate powder (CO).

Analysis of the obtained thermograms regarding physical and kneaded solid powders showed three thermal decomposition events. However, the co-evaporate showed four thermal decomposition events (Figure 4e). In addition, the physical mixture, kneaded, and the co-evaporate solid powders showed an endothermic dehydration event between 65.0 °C and 150.0 °C with a losing water mass of about 10.72%, 11.16%, and 13.19%; respectively. The second endothermic event for physical mixture and kneaded solid powder was observed between 260.0 °C and 321.0 °C with highest mass loss of 19.52% 17.66% at temperature 300.0 °C; respectively. While the second endothermic event for the co-evaporate solid powder occurred at the temperature range of 335.0 °C and 393.0 °C and had a mass loss of about 13.13%. These thermal behavior changes showed that FZ’s thermal stability was improved. Furthermore, the co-evaporate product exhibits higher thermal stability than the physical mixture, kneaded solid powder, and FZ alone [34]. The physical mixture and kneaded solid powder third endothermic event ranged between 440.0 °C and 466.0 °C at a maximum temperature value of 453.0 °C with a mass loss of 19.52% and 17.66%, respectively. On the other hand, the third co-evaporate thermal decomposition event was found at 465.0 °C in, 435.0 °C and 476.0 °C, with a mass loss of 13.3%. The fourth co-evaporate thermal degradation event was observed at 750.0 °C in 735.0 °C and 766.0 °C, with a mass loss of 10.69%. The obtained results found that the co-evaporate product showed the lowest mass loss in the range above 400 °C, which indicated that the FZ molecules replaced most of the solvent molecules in the sulfonatocalix[4]naphthalene cavity during the complexation process [34,66].

The TGA thermograms analysis results for physical mixture and kneaded powders revealed that the endothermic melting point peaks related to the FZ and the sulfonatocalix[4]naphthalene shifted to new positions and showed alteration in peaks shape in comparison with pure FZ and sulfonatocalix[4]naphthalene. These results confirmed the formation of the partially amorphous compound with lower crystallinity due to the partial complexation between FZ and they investigated host molecules in both kneaded powder and physical mixture. On the other hand, the co-evaporate product increased the observed peak number compared to pure FZ and host concomitant, broadening and shifting the peaks to new positions. These findings supported the creation of an inclusion complex between FZ and the examined host molecule and the production of highly amorphous material in the co-evaporation product. Similar findings suggested changes in one or more of the following parameters: the location, shape, and area of the guest peaks, as well as an increase or reduction in the number of guest peaks, corroborate the creation of an inclusion complex between the host and guest molecules [42,55,65,67]. The obtained results also come in agreement with the DSC results.

#### 2.2.3. Powder X-ray Diffractometry (PXRD)

**Figure 5:**
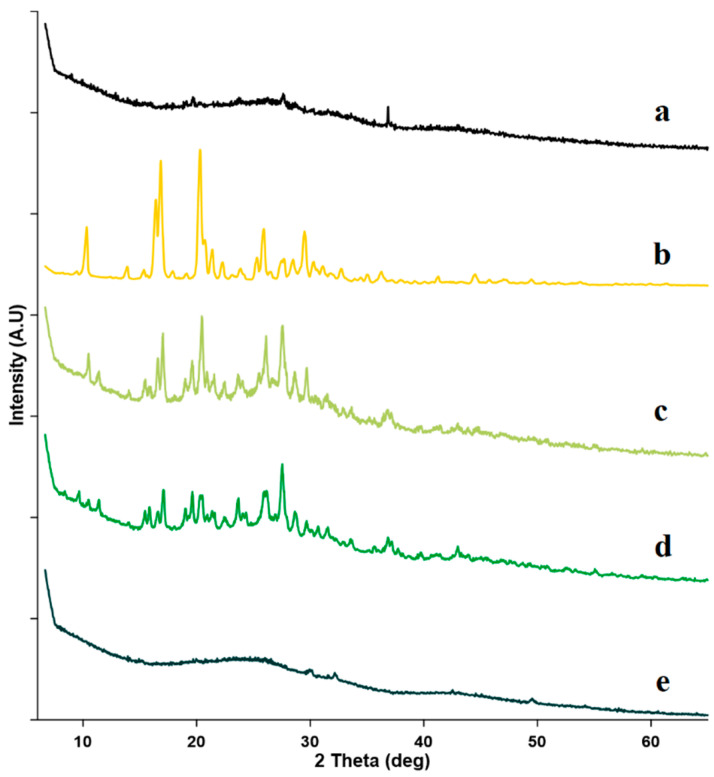
PXRD for: (**a**) Sulfonatocalix[4]naphthalene, (**b**) fluconazole (FZ) (**c**) Physical mixture (PM) (**d**) Kneaded (KN) and (**e**) co-evaporate powder (CO).

The PXRD plays a significant role in identifying the state of solid materials, whether it has a crystalline or an amorphous state [68,69]. In addition, it also reveals the chemical composition of crystalline materials and other structural parameters [68]. Figure 5 depicts the PXRD pattern of FZ, sulfonatocalix[4]naphthalene, and their equivalent 1:1 molar ratio powdered systems. The X-ray diffraction pattern of sulfonatocalix[4]naphthalene showed no diffraction peaks, indicating its amorphous nature (Figure 5a). While, pure FZ drug had multiple distinct high-intensity peaks at 2 diffraction angle of 10.5 (2222), 14.0 (774), 16.5 (3175), 17.3 (4523), 20.5 (4921), 21.0 (1786), 21.5 (1389), 22.0 (1440), 23.5 (952), 26.5 (2143), 27.5 (1071), 28.0 (1052), 29.0 (2024), 31.0 (992), 31.3 (774), 33.0 (714), 36.0 (635), 41.0 (635), 44.0 (437), and 49.5 (357) demonstrating its crystalline nature (Figure 5b) [32,70]. The PXRD patterns of the physical mixture and kneaded solid powder revealed the crystalline character of FZ, although they became broader and were reduced in their intensity. The physical mixture showed several peaks at 2θ diffraction angle of 10.5 (288), 16.5 (535), 17.1 (640), 20.5 (721), 26.5 (395), 27.5 (686), 28.0 (488), 29.0 (500), and 36.0 (279). Also, kneaded solid powder presented several peaks at 2θ diffraction angle of 17.3 (523), 20.0 (521), 21.0 (512), 23.5 (477), 26.0 (500), 27.5 (614), 28.0 (410), 37.0 (295), and 43.0 (250). It was noticed from PXRD results that the kneaded solid powder has broader and lower-intensity peaks compared with these obtained from the physical mixture. The presence of low intensity and broad FZ diffraction peaks in both physical mixture and kneaded solid powders could be attributable to the dilution effect of the carrier [71] and possible interaction between the FZ and the host. Many researchers also reported a similar finding, they reported the reduction and broadening of the PXRD peaks occurred due to the interaction between the guest and the host [42,72,73].

PXRD analysis of co-evaporate revealed a perfect lack of FZ crystalline peaks. This discovery showed that FZ had been completely transformed into an amorphous form (Figure 5e), confirming the complete inclusion of FZ into sulfonatocalix[4]naphthalene large-deep cavity. This result was in good agreement with similar reported findings by other researchers [72,74,75] and the obtained DSC results.

#### 2.2.4. Fourier Transform Infrared Spectroscopy

**Figure 6:**
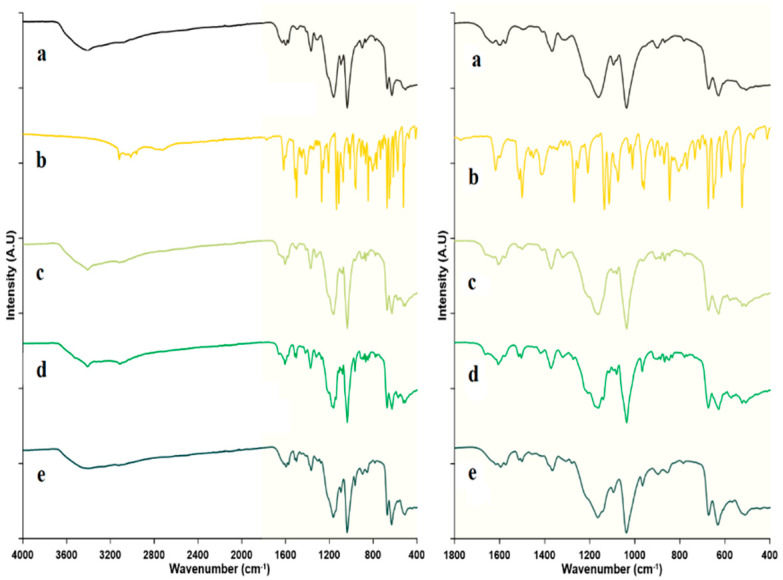
FT-IR spectra for: (**a**) Sulfonatocalix[4]naphthalene, (**b**) fluconazole (FZ) (**c**) Physical mixture (PM) (**d**) Kneaded (KN) and (**e**) co-evaporate powder (CO).

For further investigation of the possibility and kind of physicochemical interactions between FZ and the utilized host molecule, FT-IR spectra were obtained for pure FZ, sulfonatocalix[4]naphthalene, and their solid powders generated via physical mixing, kneading, and co-evaporate procedures. Spectrum of sulfonatocalix[4]naphthalene [29,76] exhibited characteristic broadband in the region of 2900–3600 cm^−1^, corresponding to the starching vibration of naphthalene hydroxyl groups (Figure 6a). The stretching vibration of the double bond in naphthalene aromatic rings was ascribed to the other peaks in 1632 to 1572 cm^−1^. The characteristic sulfonic acid group peak was observed at 1301cm^−1^. Also, the sulfonatocalix[4]naphthalene spectrum exhibits many distinctive peaks in 1157 to 500 cm^−1^. FT-IR spectrum of FZ [33,77] showed a broad peak in the region of 3119–2714 cm^−1^, resulting from vibration stretching of hydroxyl groups, and sharp characteristic peaks, due to the stretching vibration of triazole ring double bond at 3119 cm^−1^, 3000 cm^−1^, 1500 cm^−1^, 1419 cm^−1^, 1133 cm^−1^, and 958 cm^−1^. In addition, sharp peaks at 1620 cm^−1^ and 1512 cm^−1^ are assigned to the stretching aromatic double-bound. Also, C**^…^**F bound stretching appeared at 1271cm^−1^, propane backbone stretching vibration presented at 1419 cm^−1^, and 1115 cm^−1^ and C**^…^**OH, bound banding and stretching vibration appeared at 1272 and 1072 cm^−1^, respectively (Figure 6b). The FT-IR spectra of the physical mixture and kneaded solid powder showed broadening, a significant reduction, and shifting of the characteristic peaks of the FZ (Figure 6c,d). The stretching vibration of triazole rings double bond at 3119 cm^−1^ shifted to 3405 cm^−1^ and 3357 cm^−1^. Also, the stretching vibration peak at position 3000 cm^−1^ shifted to 3258 cm^−1^ and 3071 cm^−1^ for physical and kneaded products, respectively. It was also noticed that the FT-IR spectra for all prepared solid powders have a significant reduction in the peak intensity at positions 1512 cm^−1^ and 1500 cm^−1^. Furthermore, the FT-IR spectra of the physical mixture and kneaded powder exhibited a significant reduction and shifting of the peak intensity at 1419 cm^−1^ to 1417 cm^−1^ and 1415 cm^−1^, respectively. Those results could be attributed to the partial inclusion of the FZ in the host cavity and partial amorphization of the crystalline FZ [42,52,74].

The FT-IR spectrum of the co-evaporate product exhibited the disappearance of the FZ peaks located at 3119 cm^−1^, 3000 cm^−1^, and 1419 cm^−1^ (Figure 6e), which might be confirmed the formation of FZ-sulfonatocalix[4]naphthalene inclusion complex [42]; while the intensity of the rest of the FZ characteristic peaks was reduced. Also, the characteristic bands of the sulfonatocalix[4]naphthalene in the co-evaporate product shifted to the new positions of 1590 cm^−1^, 1361 cm^−1^, 1168 cm^−1^, and 1036 cm^−1^ compared with pure sulfonatocalix[4]naphthalene. These behaviors could be strongly supported by forming an inclusion complex with some new bands. A similar reported finding states that the disappearance and changes in the peak intensity or position confirmed the formation of inclusion complexes [42,52,74]. In addition to that, the DSC, TGA, and PXRD results confirmed this finding. On the other hand, these observations might be attributable to the possibility of non-covalent intermolecular interactions such as the strong H**^….^**F hydrogen bonding and π-π intermolecular interactions between the FZ benzene ring with hydroxyl groups located in the deep and large cavity of the sulfonatocalix[4]naphthalene [34].

### 2.3. Dissolution Test

**Figure 7:**
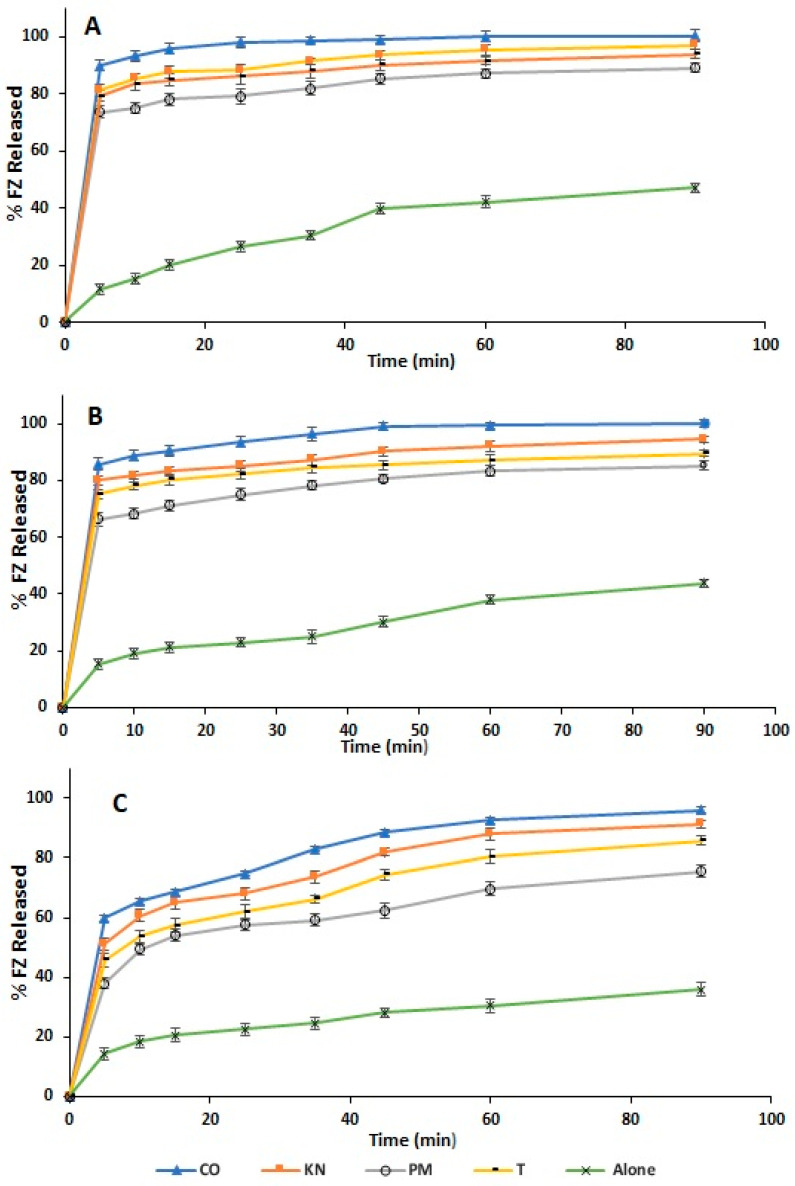
In vitro dissolution performance of the FZ to the differently prepared systems; co-evaporate (CO), kneaded (KN), physical mixture (PM), and FZ alone (FZ); in (**A**). pH 1.2, (**B**). phosphate buffer pH 7.4; (**C**). distilled water; Key: CO (filled triangle), KN (Filled square), PM (empty circle), and FZ alone (star); (*n* = 3, ±S.D.).

The pharmaceutical solid dosage’s dissolving performance and solubility are the most important physicochemical factors influencing the drug’s bioavailability [59]. The effect of sulfonatocalix[4]naphthalene as a host on FZ dissolving performance was investigated In vitro. Figure 7 illustrated the dissolution profiles of all the prepared solid powders, including physical mixture, kneaded, and co-evaporate powders, besides pure FZ at different investigated media, pH 1.2, phosphate buffer of pH 7.4, and distilled water at 37 ± 0.5 °C. Dissolution studies of the pulverized FZ–sulfonatocalix[4]naphthalene complexes significantly improved the dissolution rate compared with untreated FZ. Initially, FZ showed a similar dissolution profile at pH 1.2 and 7.4 from the different prepared solid powders with relatively higher dissolution rates at pH 1.2. This could be attributed to the enhanced solubility of FZ in the acidic medium, as indicated by the solubility study. In addition, the weak basicity-acidity nature of FZ and a strong interaction between the FZ and host [30,56] could also be responsible for this behavior. The co-evaporate product in pH 1.2, phosphate buffer pH 7.4, showed a fast initial dissolution rate of more than 87 ± 1.87% of FZ released within the first 10 min. The percentage of FZ dissolved from co-evaporate, kneading, physical mixture, and FZ alone at pH 1.2 after 5 min was 89.86 ± 1.89%, 81.22 ± 2.00%, 73.56 ± 1.85%, and 11.55 ± 1.80%. Also, the percentage of the FZ dissolved at pH 7.4 at the same time was 85.76 ± 2.03%, 80.04 ± 1.61%, 66.35 ± 2.38%, and 15.26 ± 2.01%, respectively. On the other hand, the percentage of FZ dissolved in distilled water after 5 min was 59.8 1 ± 2.16%, 51.23 ± 1.57%, 37.90 ± 1.88%, and 14.40 ± 2.06%; respectively. According to those results, it is evident that the co-evaporate product exhibited a faster and higher dissolution rate in the investigated dissolution media. The observed improvement in FZ dissolving performance in the co-evaporate product is attributed to creating the FZ-host inclusion complex and drug amorphization [42,52,74]. Whereas, improved dissolution rate with kneaded and physical mixture could be possibly attributed to the wetting effect [78] of sulfonatocalix[4]naphthalene, lowering of drug crystallinity, and increasing FZ solubility was confirmed by phase solubility results.

The improved FZ dissolution due to the inclusion complex with sulfonatocalix[4]naphthalene has a superior role in improving the physicochemical and biopharmaceutical properties of the FZ. The Higher dissolution rates are also responsible for increased absorption, bioavailability, and, as a result, faster and more effective action, which is indicated for many fungal infections.

The dissolution efficiencies (ED_25_) after 25 min [79] and relative dissolution rate (RDR_10_) [67] during the first 10 min are presented in Table 2 for the prepared solid powders. Results revealed a higher improvement in dissolution efficiency (ED_25_) and the relative dissolution rate (RDR_10_) for all the prepared solid powders than pure FZ in all investigated media. Moreover, the co-evaporate product has the highest ED_25_ with average values of 32.02 ± 0.57, 30.64 ± 0.63, and 23.95 ± 0.71 at pH 1.2, phosphate buffer of pH 7.4, and in distilled water, respectively. In addition, it has the highest RDR_10_ with average values of 6.18 ± 0.12, 4.64 ± 0.10, and 3.54 ± 0.11 at pH 1.2, pH 7.4, and in distilled water; respectively; unlike kneaded, physical mixture, and untreated FZ (Table 2). It was also noticed from the dissolution results that the highest dissolution efficiency (ED_25_), increasing folds in the dissolution rate, and RDR_10_ for the prepared solid systems were found at pH 1.2, followed by pH 7.4, and distilled water. This result also comes following that obtained from the solubility study as mentioned before. The enhancement of the FZ’s dissolution rate depended on the medium pH and the degree of interaction between FZ and sulfonatocalix[4]naphthalene [80].

The statistical analysis of the in vitro dissolution study was performed to compare the dissolution rate behaviors of the FZ-host products with pure FZ in different investigated media using one-way between-subjects ANOVA. The dissolution data were statistically analyzed using a one-way between-subjects ANOVA to determine the influence of the medium on dissolving performance. The null hypothesis (H_o_) assumes no substantial variation in the proportion of drug release in the various mediums at pH 1.2, 7.4, and distilled water by using the same technique at *p* ≤ 0.05. As a result, there is a statistically significant difference in the means dissolution rate in the various dissolution media. The null hypothesis was rejected, and the alternative hypothesis was accepted due to the *p*-value ≤ 0.05. To identify the medium, comparisons using the Bonferroni post hoc test were performed. The Bonferroni post hoc test revealed a significant mean difference in the percentage of drug release in the various dissolving media utilized. As a result, the following proportion of medications was released: pH 1.2 > phosphate buffer pH 7.4 ≈ distilled water. In addition, a one-way between-subjects ANOVA for the dissolution rate was performed. The null hypothesis (H_o_) states that there is no significant difference between the mean dissolution rate of the co-evaporated, kneaded, and physical combination and the mean dissolving rate of pure FZ in the same medium at *p* ≤ 0.05. The null hypothesis (H_o_) was accepted, implying that the proportion of drugs released by co-evaporate, kneaded, or physical mixture methods are unaffected by the kind of dissolving medium.

### 2.4. Antimycotic Activity

**Figure 8:**
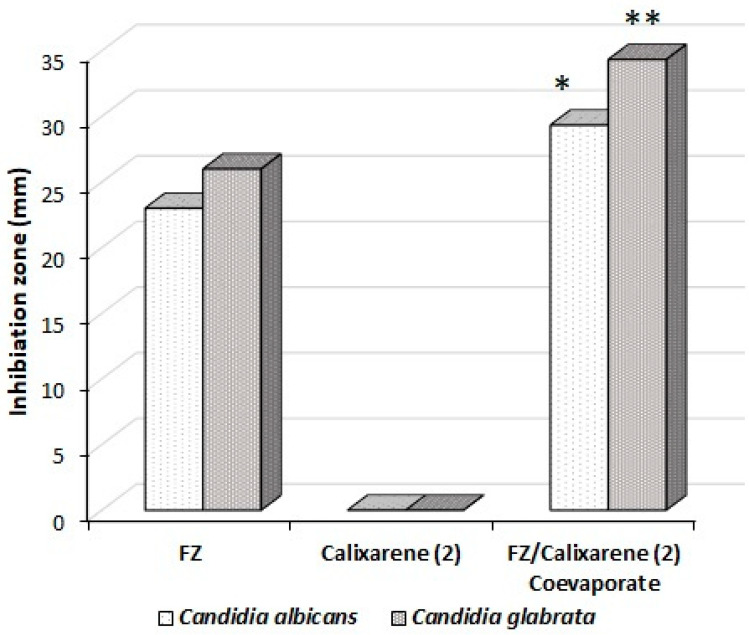
The antimycotic activity of pure FZ, sulfonatocalix[4]naphthalene, and co-evaporate inclusion complex of FZ-sulfonatocalix[4]naphthalene (conc.; 6 mM) against pathogenic clinical strains of *Candida albicans* and *Candida glabrata*. Inhibition zones were measured in mm. * FZ/co-evaporate significantly (*p* ≤ 0.05; *t*-test) active against *Candida albicans* than pure FZ; ** FZ/co-evaporate significantly (*p* ≤ 0.05; *t*-test) active against *Candida glabrata* than pure FZ; (*n* = 3, ±S.D.).

**Figure 9:**
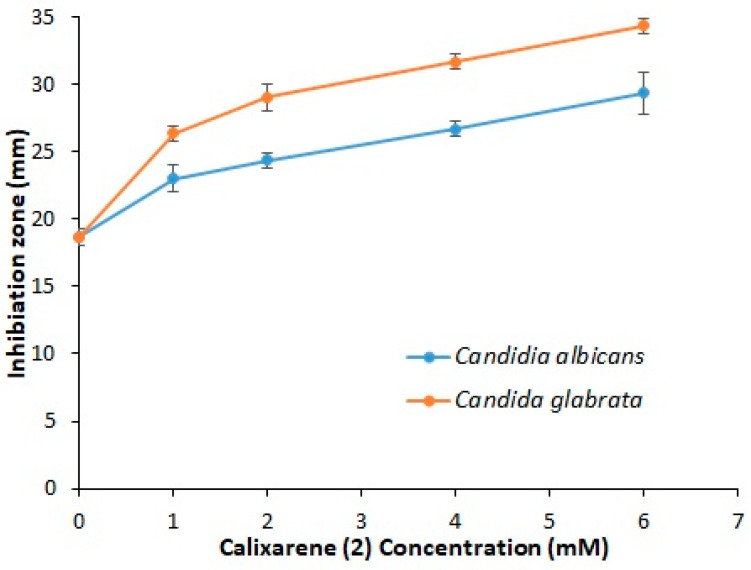
Effect of different concentrations of sulfonatocalix[4]naphthalene on inhibition zones of FZ against clinically pathogenic strains of *Candida albicans* and *Candida glabrata*; (*n* = 3, ±S.D.).

The antimycotic effect of the co-evaporate powder of inclusion complex of FZ-sulfonatocalix[4]naphthalene, FZ, and sulfonatocalix[4]naphthalene solutions was evaluated based on inhibition zones of the tested fungi species, *Candida albicans* and *Candida glabrata* using agar cup diffusion method as previously reported [81]. Inhibition zones of the tested powders are presented in Figure 8 and Figure 9. FZ showed an antimycotic activity against both *Candida albicans* and *Candida glabrata* when tested at 6 mM (concentration was chosen from the phase solubility diagram) with mean inhibition zones diameters of 23 ± 1.0 and 26 ± 1.0 mm; respectively. However, when tested at the same concentration, sulfonatocalix[4]naphthalene did not show any antimycotic activity [34]. Co-evaporate powder of inclusion complex of FZ-sulfonatocalix[4]naphthalene showed a significantly higher (*p* ≤ 0.05, *t*-test) antimycotic activity compared with FZ alone against both fungi species. In Addition, it also showed a significantly (*p* ≤ 0.05, *t*-test) higher antimycotic activity against *Candida glabrata* compared with *Candida albicans* (Figure 8). The concentration of sulfonatocalix[4]naphthalene increases, and the inhibition zones of both tested fungi species increase (Figure 9). This might be due to the inclusion complex’s formation increasing the solubilized quantities of FZ, boosting drug diffusion into the agar media. These results are consistent with the phase solubility diagrams examined. FZ was shown to be more soluble when sulfonatocalix[4]naphthalene was used at a concentration of 6 mM. It was also intriguing to discover that the acquired outcomes were more promising than those obtained from Abranches et al. [34], who found that the complexed FZ with sodium *p*-sulfonatocalix[4]arene did not improve significantly the efficacy of FZ against *Candida albicans* and *Candida glabrata* non-clinical strains.

## 3. Materials and Methods

### 3.1. Chemicals

The Jordanian Pharmaceutical Manufacturing (JPM) group, Amman-Jordan, donated Fluconazole. Chromotropic acid disodium salt dehydrates (Sigma-Aldrich, St. Louis, MO, USA), acetone (Tedia, Fairfield, OH, USA), methanol (Fischer), hydrochloric acid, potassium dihydrogen (AZ Chem., Pretoria, South Africa), dipotassium hydrogen phosphate (PRS, Paneac, Barcelona, Spain). Pathogenic clinical strains of *Candida albicans* and *Candida glabrata* were obtained from Assiut University Hospital. Strains are further maintained at the laboratory of Moubasher Mycological Center, Assiut University. Sabouraud dextrose agar was obtained from SDA, Difco Laboratories, Detroit, MI, USA. The other chemicals used in this study were of analytical grades.

### 3.2. Phase Solubility Test

The sulfonatocalix[4]naphthalene has been synthesized according to the reported literature procedure [27] and used after recrystallization by using the mixed solvent system by dissolving it in a minimum amount of hot water. Ethanol was added gradually until forming turbidity; then, the mixture was heated again until boiling. The Higuchi and Connors solubility method was used to investigate the increasing sulfonatocalix[4]naphthalene concentration on the solubility of FZ [57]. To summarise, excess FZ powder was introduced to a series of vials containing escalating concentrations of sulfonatocalix[4]naphthalene (0.0 to 20.0 mM) and 10 mL of distilled water or various pH solutions 1.2 and 7.4, which mimicked the stomach and intestine pH conditions. Samples were prepared in triplicate with FZ: sulfonatocalix[4]naphthalene at molar ratio of (1:0, 1:0.2, 1:0.4, 1:0.6, 1:0.8, 1:1) for each different solubility medium. Further, the screw-capped glass vials were shaken (Shaker-Memmert-GmbH, Schwabach, Germany) for four days at a constant temperature of 25 ± 0.5 °C, after which equilibrium was attained [53]. Then, the obtained suspensions were centrifuged (Boeco-Germany) for 20 min at 3000 rpm, and a 0.45 μm syringe (syringe Filter PTFE, Santa Cruz Biotechnology, Inc., Dallas, TX, USA) membrane filter was used to filter the supernatant. After that, UV/VIS double beam spectrophotometer SCOTech SUP-26 (Sco Tech., Gmbh, Dingelstadt, Germany) was used against blanks to dilute and measure the clear filtrate solution at λ_max_ of 260 nm, which was prepared as the same concentration of sulfonatocalix[4]naphthalene at three different types for avoiding any interferences. The stability constant (*Ks*) of the FZ complexes was calculated from the phase-diagrams slope using the Equation (1) below [82].

(1) $$Ks=slop/S_{o}\left(1-slop\right)$$

where, slope value is obtained from plotting the concentration of FZ versus the concentration of sulfonatocalix[4]naphthalene, *S_o_* is the solubility of FZ alone without a host in the different investigated media. Solubility measurements were calculated in triplicate and presented as a mean ± SD.

### 3.3. Preparation and Confirmation of Inclusion Complexes

#### 3.3.1. Preparation of Inclusion Complexes

Different methods were adopted to prepare solid complexes of FZ with sulfonatocalix[4]naphthalene. In all methods, the molar ratio was maintained at 1:1 between FZ and sulfonatocalix[4]naphthalene, depending on the result obtained from phase solubility studies to prepare solid complexes. The first is the physical mixture prepared from the previously sieved host and FZ powder through mesh no. (150 µm) by blending for 20 min using a spatula and mortar. The second method, kneading powder, was prepared by slowly adding FZ powder to the previously mixed sulfonatocalix[4]naphthalene with a few drops of water, forming a homogeneous paste in a mortar. Then, a few drops of water were added to the mixture, and it was kneaded for a further 20 min. The resulting paste was dried in a vacuum oven Thermo Stable OV-30, Wonju-si, Korea) for 24 h at 40 °C and then sieved through mesh no. 150 µm.

The third method is solvent evaporation, prepared in a round bottom flask by dissolving FZ powder and sulfonatcalix[4]naphthalene in 3 mL methanol and 20 mL distilled water; respectively. The mixture was stirred thoroughly at 25 °C for 1 h, then a rotary evaporator (Buchi, Meierseggstrasse, Switzerland) was used to remove the solvent at 40 ± 0.5 °C. The obtained paste was dried in a vacuum oven (Thermo Stable OV-30, Wonju-si, Korea) for 24 h at 40 °C. The solid powder was ground into a fine powder and sieved through a screen of 150 µm. For conducting further analysis, all solid complexes were kept in a desiccator over anhydrous CaCl_2_.

#### 3.3.2. Confirmation of Inclusion Complex

##### Differential Scanning Calorimetry (DSC)

A Shimadzu DSC-50 differential scanning calorimeter (Shimadzu Corporation, Tokyo, Japan) was used to conduct all samples’ DSC thermograms. Approximately 2–5 mg of the powder samples were added to 50 μL aluminum pans with a thickness of 0.1 mm. The pans were then covered with a 0.1 mm thick aluminum lid, and an empty pan served as a reference. The DSC samples were heated from ambient temperature to 850 °C at a rate of 10 °C/min with 40 mL/min of nitrogen flow rate. The DSC instrument temperature was calibrated using indium metal.

##### Thermogravimetric Analysis (TGA), Derivative Thermogravity (DTG)

The thermoanalyzer Jupiter STA 449 F5 (Netzsch, Germany) was used to conduct a thermogravimetric analysis of all samples. Samples were placed in aluminum oxide (Al_2_O_3_) crucible, then heated to 1050 °C, with a heating rate of 35 °C/min under a nitrogen atmosphere. The curves were examined using the Netzsch Proteus Analysis Software.

##### Powder X-ray Diffraction (PXRD)

Philips 1710 powder diffractometer instrument with Cu Kα radiation (1.54056 Å) was used to perform the X-ray diffraction patterns of the prepared samples. The instrument was previously calibrated by polycrystalline silicon standard. A Cu target tube with a voltage of 40 kV and a current of 40 mA was used, and a single crystal graphite monochromator. The scanning speed was set to 0.6°/min, and the wide-angle diffraction was adjusted at 4° < 2θ < 60°.

##### Fourier Transform Infrared Spectroscopy (FT-IR)

The FT-IR samples were crushed and mixed with 400 mg of KBr salt using a mortar and pestle, compressed, and examined using Bruker Vertex 80v ATR-FTIR equipment. The spectra were recorded from 4.000–400 cm^−1^ spectra range with 512 scans and a resolution of 4 cm^−1^.

### 3.4. Dissolution Test

In vitro dissolving behaviors of pure FZ were compared to those of their solid complexes. The dissolving rate investigations were carried out in a 500 mL dissolution medium of varied pH values, pH 1.2, phosphate buffer of pH 7.4, and distilled water (DW) using a dissolution tester of USP type–II (Paddle) (Pharma test PTWS 820D, Hainburg, Germany). The temperature was maintained at 37 ± 0.5 °C, and the stirring speed was set at 100 ± 1.0 rpm. In the dissolving media, 75 mg of FZ samples were scattered. The samples of 5mL were taken at 5; 10; 15; 25; 35; 45; 60; 90 min; at the same time, 5 mL solution should be replaced. Then the samples were filtered using 0.45 μm filters (Syringe Filter PTFE, Santa Cruz Biotechnology, Inc.). They were diluted adequately whenever necessary, and the dissolution medium as blank at λ_max_ of 260 nm was set for analysis by UV-VIS spectrophotometer. The dissolution experiments were carried out in triplicate for all the samples, and the mean values were listed. The dissolution efficiency (DE) was calculated according to Equation (2) reported by Khan [79].

(2) $$DE_{t}=\frac{\int_{0}^{t}y\times d_{t}}{y_{100}\times t}\times 100$$

where; the *DE_t_* is the calculated area from time zero up to time *t*, under the dissolution curve; *t*, is the total time needed for the drug released, *y* is the percentage of the drug released at the time *t*, and *y*_100_ is considered a 100% of FZ released.

### 3.5. Antimycotic Activity

The antimycotic activity of FZ and co-evaporate product of inclusion complex FZ with sulfonatocalix[4]naphthalene in different concentrations used in the constructed phase solubility diagram, FZ alone and sulfonatocalix[4]naphthalene alone was determined using a plate micro-bioassay, agar cup diffusion method. The preparation of the agar medium involved dissolving 65 g of sabouraud dextrose agar powder in a liter of distilled water, and sterilization was carried out through autoclaving for 20 min at 121 °C. Before testing, pathogenic strains of both *Candida albicans* and *Candida glabrata* were inoculated into liquid sabouraud’s dextrose medium for 24 h. The fungi suspension’s turbidity is matched with standard 0.5 M McFarland barium sulfate indicating approximately 10^7^ viable cells/mL [83]. In agar medium, *Candida* strains were seeded to a concentration of 10^5^ viable cells per ml from standardized suspension. Petri plates were filled with seeded agar media (11 cm width). A borer was used to cut three wells in each dish. Each well contained a 50 µL sample of tested sample solutions. After resting the Petri-dishes for 1 h, they were incubated for 20 h at 32 °C. Then, measurements were taken of the inhibition zone diameters of both strains of *Candida* in triplicate.

### 3.6. Statistical Analysis

The one–way analysis of variance (ANOVA) using software (SPSS version 23) was applied to estimate dissolution rate parameters. This was followed by multiple comparisons Bonferroni post hoc test. The statistical analysis findings were considered significant if the *p*-value was less than 0.05. The mean and standard deviation of all values is given. In addition, the antimycotic activity for the investigated powders was tested using the student *t*-paired test. The significance of variance was tested using the *F*-test.

## 4. Conclusions

Sulfonatocalix[4]naphthalene could be a promising complexing agent to enhance FZ’s aqueous solubility and dissolution performance. The solubility of the FZ has improved by about 31 at pH 1.2 and 25 folds at the phosphate buffer of pH 7.4. FZ can form inclusion complexes with sulfonatocalix[4]naphthalene in a 1:1 molar ratio, resulting in A_L_ type phase-solubility diagrams in the investigated media. Inclusion complexation of the drug with sulfonatocalix[4]naphthalene was evident via the co-evaporate technique compared with the other methods, as proofed by the studied physicochemical analysis tools DSC, TGA, PXRD, and FT-IR. Furthermore, the preparation processes impacted the FZ dissolving performance but not significantly by the medium employed in the dissolution investigation. As a result, the highest dissolution efficiency (ED_25_) and relative rate of the dissolution (RDR_10_) with average values of 32.02 ± 0.57 and 6.18 ± 0.12, respectively, were obtained from the inclusion complex prepared by the co-evaporate method and utilizing the acidic medium (pH 1.2). The antimycotic activity follows the same pattern as the solubility study. The co-evaporate powder of FZ with sulfonatocalix[4]naphthalene showed a significantly higher (*p* ≤ 0.05, *t*-test) antimycotic activity compared with FZ alone against both investigated fungi species. In addition, it also showed a significantly (*p* ≤ 0.05, *t*-test) higher antimycotic activity against *Candida glabrata* compared with *Candida albicans*.

In conclusion, the hydrophobic FZ drug showed an increase in the dissolution rate and antimycotic activity in water-soluble sulfonatocalix[4]naphthalene as an efficient new complexing agent. Furthermore, sulfonatocalix[4]naphthalene could be an alternative to the cyclodextrin macrocycles as a drug carrier in improving the dissolution and bioavailability of poorly soluble drugs. Future studies will include attempts to use this promising drug carrier to enhance the solubility of poorly water-soluble drugs, emphasizing in vivo studies.

## Figures and Tables

**Table 1:** The phase solubility study of the Fluconazole-sulfonatocalix[4]naphthalene complex at different media.

Medium Type	Phase Solubility Diagram Type	Stability Constant ± S.D. (M^−1^)	Increasing of Solubility (S_t_/S_o_)
0.1N HCl pH 1.2	A_L_	2861.112 ± 0.095	31.103
Phosphate buffer of pH 7.4	A_L_	1881. 830 ± 0.012	25.241
Distilled water	A_L_	501.266 ± 0.011	18.500

S_t_ solubility of FZ in sulfonatocalix[4]naphthalene solution; S_o_, the solubility of FZ in distilled water.

**Table 2:** The Dissolution Efficiency (ED_25_) and Relative Dissolution rate (RDR_10_) in different media for the co-evaporate (CO), kneaded (KN), physical mixture (PM), and fluconazole alone; (*n* = 3, ±S.D.).

Methods	ED_25_ ± S.D., *n* = 3			Fold Increase			RDR_10_		
	pH 1.2	pH 7.4	D.W	pH 1.2	pH 7.4	D.W	pH 1.2	pH 7.4	D.W
CO	32.02 ± 0.57	30.64 ± 0.63	23.95 ± 0.71	4.30	4.25	3.41	6.18	4.64	3.54
KN	29.18 ± 0.50	27.99 ± 0.56	21.88 ± 0.64	3.92	3.88	3.11	5.66	4.26	3.28
PM	25.95 ± 0.72	24.23 ± 0.51	17.99 ± 0.64	3.48	3.36	2.56	4.95	3.54	2.67
FZ powder	7.45 ± 0.64	7.20 ± 0.62	7.02 ± 0.70	-	-	-	-	-	-

ED_25_ dissolution efficiency; RDR_10_ relative dissolution rate; D.W distilled water.

## Data Availability

Not applicable.

## Sample Availability

Not available.
